# A circus postcard showing short statue in a clown and a horse

**DOI:** 10.1007/s40618-021-01654-w

**Published:** 2021-08-07

**Authors:** W. W. de Herder

**Affiliations:** Department of Internal Medicine, Sector of Endocrinology, Rg520, Erasmus MC, Dr. Molewaterplein 40, 3015 GD Rotterdam, The Netherlands

**Keywords:** Achondroplasia, Circus, Short stature, Horse

**Fig. 1:**
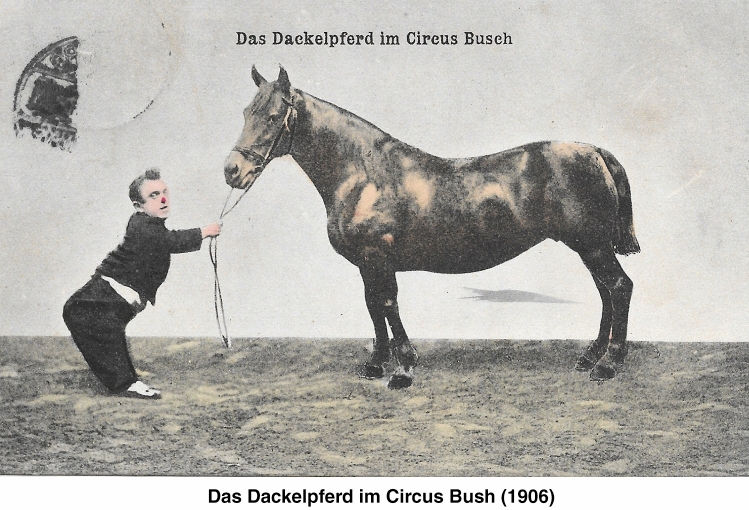
Das Dackelpferd im Circus Bush (1906). Picture from the collection of W.W. de Herder

A picture postcard of the German Zirkus Busch (the Busch Circus) dating 1905–6 titled: "Das Dackelpferd im Circus Bush" = "The dachshund-shaped horse in circus Bush" showing a male with typical achondroplasia dressed as a clown and a dysmorphic Friesian horse with short limbs (Fig. 1).

The famous German Bush Circus was founded in 1884 by Paul Vinzenz Theodor Busch (1850–1927). It was a travelling circus with its main circus building, which could hold up to 4300 spectators, established in Berlin in 1895.

Achondroplasia is the most common cause of short statue in humans and affects about 1 in 25,000 people. Achondroplasia is inherited as an autosomal dominant trait. However, about 80% of cases result from a de novo mutation. It is caused by gain-of-function variant in the FGFR3 gene. Its predominant phenotype is: disproportionate short stature with rhizomelic shortening of the arms and the legs, brachydactyly, kyphoscoliosis and accentuated lumbar lordosis, macrocephaly, frontal bossing, midface retrusion, and saddle nose deformity.

In Friesian horses, short statue is characterized by the limbs being 25% shorter than normal and growth retardation of the ribs. Usually, the head and back grow faster than the limbs and ribs giving these horses the characteristic disproportional appearance. Furthermore, their bodyweight is reduced by 50%. The estimated incidence of this disorder is 1:400. It is assumed that this disorder is inherited as an autosomal recessive monogenic trait [1].
